# Testicular infarction secondary to protein S deficiency: a case report

**DOI:** 10.1186/1471-2490-6-17

**Published:** 2006-07-07

**Authors:** Damian McKay, Conor Marron, Robin Brown

**Affiliations:** 1 The Department of Surgery, Daisy Hill Hospital, Newry, Co Down, BT35 8DR, N. Ireland, UK; 2 C/O The Department of Surgery, The Institute of Clinical Science, The Royal Victoria Hospital, Belfast, BT12 6BA, N. Ireland, UK

## Abstract

**Background:**

Protein S deficiency is an inherited cause of thrombophilia. We present the second reported case in the literature of a man developing testicular infarction secondary to protein S deficiency.

**Case presentation:**

A 63 year old man presented with sudden onset of pain in his left hemi-scrotum. Despite oral warfarin therapy the plasma INR was only 1.4 at presentation. Doppler ultrasound scan of the scrotum confirmed absent blood flow to the left testis with increased echogenicity. Orchidectomy was performed to remove the necrotic testis. Post-operatively the patient did well and was referred to the Regional Haemophilia and Thrombosis Centre for further management.

**Conclusion:**

The case highlights a rare but potential complication of protein S deficiency and demonstrates the importance of adequate anticoagulation in these patients.

## Background

Protein S deficiency is a rare blood disorder which is a risk factor for thrombophilia. It affects around 1 in 20000 of the population [1]. It may be hereditary or acquired. Hereditary protein s deficiency is divided into three types: Type 1, Type 2a and Type 2b.

The most common cause of acquired protein S deficiency is pregnancy. Acquired protein S deficiency may be seen in some patients who have developed disseminated intravascular coagulation (DIC), deep vein thrombosis (DVT) or pulmonary embolism (PE). The treatment of protein s deficiency is systemic anticoagulation with warfarin or a similar agent. Testicular infarction is a rare but recognised complication of thrombophilia.

## Case presentation

A 63 year old man presented to his General Practitioner with a history of left sided testicular pain for 24 hours. The pain had increased with time. He was referred to the Accident and Emergency Department of our hospital where he was examined and an ultrasound scan arranged. He had no urinary symptoms. He was currently under the care of the haematology department with protein S deficiency. He had a history of DVT and PE in 1965 and received anticoagulation therapy with warfarin for a year. The diagnosis of protein S deficiency was made in 1993, after investigation for recurrent leg ulceration, and therapy with warfarin and subsequently low molecular weight heparin commenced. At presentation his International Normalised Ratio (INR) was sub-therapeutic at 1.4, despite daily oral administration of 6 mg of Warfarin daily. The patient also self administered 1 mg/kg of enoxaparin since diagnosed in 1993. The patient had neither previous cardiovascular history nor clinical evidence of atrial fibrillation.

Ultrasonography of the left testis demonstrated increased echogenicity around the head of the epididymus in particular, suggesting haematoma. The echogenicity of the testis was abnormal and no blood flow was demonstrated on Doppler interrogation. At surgery a left groin incision was made with the cord was clamped near the internal ring. The testis and epididymus were haemorrhagic and necrotic and there was no evidence of torsion.

Histological examination revealed a necrotic testis. The blood vessels were congested with evidence of thrombus. No obvious emboli were present. There was no evidence of carcinoma. Post surgery the patient recovered well and was commenced on clopidogrel 75 mg and enoxaparin 100 mg daily. He was referred to the Regional Haemophilia and Thrombosis Centre for further management. He has since been reviewed and is well.

## Discussion

We believe this to be only the second such case of testicular infarction secondary to protein S deficiency reported in the world literature [1].

Testicular infarction secondary to Protein S deficiency is exceedingly rare. The patient presents with symptoms of testicular pain. On examination, the scrotum may be red, swollen and tender and may mimic the clinical examination of a patient with testicular torsion. The pathophysiology of this clinical picture is congestion and thrombosis of the venous and arterial blood supply to the testis. Infarction of other organs such as brain, heart, spleen and more commonly recognised as sequelae of the condition.

Protein S deficiencies are associated with superficial and deep vein thrombosis and pulmonary embolism [2]. Protein S deficiency may be hereditary or acquired. The acquired state is usually due to hepatic diseases or a vitamin K deficiency. Congenital protein S deficiency is an autosomal dominant disease, and the heterozygous state occurs in approximately 2% of unselected patients with venous thromboembolism.

The management of Protein S deficiency involves initial heparinisation at the time of the initial presentation followed by commencement on warfarin. In this case the patient's INR was sub therapeutic at 1.4. Levels greater than 2.0 are required to provide adequate anticoagulation. It is surprising that his INR was low as he attended the haematology clinic every fortnight for monitoring of his INR. What is more surprising is that this event occurred despite subcutaneous enoxaparin injection. It may be that patient compliance with both oral warfarin and subcutaneous enoxaparin was poor. Warfarin-induced skin necrosis may occur in these patients on initiation of warfarin therapy. This reflects a reduction in the level of protein S before the level of prothrombin is lowered into the therapeutic range. In this condition prothrombin levels stimulate the formation of venous thrombi by an unclear mechanism. These patients usually require a change to heparin therapy [3,4].

Protein S deficiency has been associated with arterial as well as venous occlusion [5,6] and it is now suggested to be an independent risk factor for peripheral arterial insufficiency [6].

Fluctuations in protein S levels are also common including in healthy members of the population [7]. It may be that individuals are more likely to have a thrombotic event during a dip in protein S levels. Other conditions, related to thrombophilia, that have been reported as aetiological factors in cases of testicular infarction are sickle cell disease, polycythemia, trauma and epididymo-orchitis [2,8,9].

## Learning points

1. Testicular infarction is a rare complication of inadequate anticoagulation in patients with pro-thrombotic conditions.

2. Ultrasound scanning is a useful modality in confirming the diagnosis in these patients.

3. A dilemma arises in the case of a young man with thrombilia and possible testicular torsion. The history and ultrasound findings could be identical but if the aetiology was due to thrombosis would systemic anticoagulation with low molecular weight heparins be more suitable than surgery? And how would one differentiate between the two?

4. Testicular infarction due to causes other than torsion or trauma is very rare. It would be useful to screen these patients for Protein S deficiency and other pro-clotting conditions.

## Abbreviations

INR – International Normalised Ratio.

DIC – Disseminated Intra-vascular Coagulation.

DVT – Deep Vein Thrombosis.

P.E – Pulmonary Embolism.

APC – Activated Protein C.

## Competing interests

The author(s) declare that they have no competing interests.

## Authors' contributions

Damian McKay and Conor Marron reviewed the case notes, carried out the literature review and prepared the initial manuscript. Mr R Brown was the consultant surgeon looking after the patient and identified the case for publication and reviewed the manuscript prior to submission.

## Pre-publication history

The pre-publication history for this paper can be accessed here:


